# Comparative Effectiveness of Proton Therapy versus Photon Radiotherapy in Adolescents and Young Adults for Classical Hodgkin Lymphoma

**DOI:** 10.14338/IJPT-21-00011.1

**Published:** 2021-07-08

**Authors:** James E. Bates, Stephanie Terezakis, Christopher G. Morris, Avani D. Rao, Shuchi Sehgal, Rahul Kumar, Raymond B. Mailhot Vega, Nancy P. Mendenhall, Bradford S. Hoppe

**Affiliations:** 1 Department of Radiation Oncology, Winship Cancer Institute, Emory University, Atlanta, GA, USA; 2 Department of Radiation Oncology, University of Minnesota, Minneapolis, MN, USA; 3 Department of Radiation Oncology, University of Florida, Gainesville, FL, USA; 4 University of Florida Proton Therapy Institute, Jacksonville, FL, USA; 5 Department of Radiation Oncology and Molecular Radiation Science, Johns Hopkins University, Baltimore, MD, USA; 6 School of Medical Sciences & Research, Sharda University, Greater Noida, India; 7 Department of Radiation Oncology, Mayo Clinic, Jacksonville, FL, USA

**Keywords:** pediatrics, Hodgkin lymphoma, outcomes, radiation therapy, proton therapy

## Abstract

**Purpose:**

Early stage (stages I-II) classical Hodgkin lymphoma (cHL) is a highly curable disease typically diagnosed in adolescents and young adults (AYAs). Proton therapy can also reduce the late toxicity burden in this population, but data on its comparative efficacy with photon radiotherapy in this population are sparse. We assessed outcomes in AYAs with cHL in a multi-institution retrospective review.

**Materials and Methods:**

We identified 94 patients aged 15 to 40 years with stages I and II cHL treated with radiotherapy as part of their initial treatment between 2008 and 2017. We used Kaplan-Meier analyses and log-rank testing to evaluate survival differences between groups of patients.

**Results:**

A total of 91 patients were included in the analysis. The 2-year progression-free survival (PFS) rate was 89%. Of the 12 patients who experienced progression after radiotherapy, 4 occurred out-of-field, 2 occurred in-field, and 6 experienced both in- and out-of-field progression. There was no significant difference in 2-year PFS among AYA patients by radiotherapy dose received (≥ 30 Gy, 91%; < 30 Gy, 86%; *P* = .82). Likewise, there was no difference in 2-year PFS among patients who received either proton or photon radiotherapy (proton, 94%; photon, 83%; *P* = .07).

**Conclusion:**

Our cohort of AYA patients had comparable outcomes regardless of radiotherapy dose or modality used. For patients with significant risk of radiation-induced late effects, proton therapy is a reasonable treatment modality.

## Introduction

Early stage classical Hodgkin lymphoma (cHL) is a highly curable disease that is most prevalent among adolescents and young adults (AYAs) [1]. Treatment of these patients can be challenging because they often straddle the age between “pediatric” and “adult.” Although modern trials in cHL share many similarities, typically including multiple cycles of anthracycline-based chemotherapy with therapy adapted per positron emission tomography (PET) response, trials straddling this age range recommend a wide variety of radiation (RT) doses. Both recently published Children's Oncology Group studies for early stage pediatric HL recommend a dose of 21 Gy at 1.5 Gy/fraction for patients requiring RT [2, 3]. Although the German Hodgkin Study Group HD10 allowed for 20 Gy of RT in very low-risk patients, other trials in adult patients typically recommend fractionated doses of ≥ 30 Gy [4–7]. Beyond that, chemotherapy regimens typically differ between pediatric and adult trials [8].

Determining the optimal treatment protocol is critical given the late effects that can arise even at low doses of anthracycline-containing chemotherapy or RT (such as second cancers, cardiac disease, and lung disease) [9–12]. Because of these concerns for late effects, proton therapy has been used in the treatment of these patients with an aim toward reducing RT doses to healthy tissues [13, 14]. However, little comparative data exist in the AYA population to establish the efficacy of proton therapy in these patients.

We describe the outcomes and patterns of failure, as well as compare outcomes, after doses above or below 30 Gy and by RT modality in a contemporary cohort of AYA patients with early stage cHL treated with RT as part of their initial therapy.

## Materials and Methods

We obtained institutional review board approval at each of the 2 institutions from which patients were included for this study: the University of Florida and Johns Hopkins School of Medicine. We then abstracted relevant demographic, treatment-related, and outcome variables for patients aged 15 to 40 years old at time of diagnosis with stages I and II cHL. All patients had histologically confirmed cHL. Pretreatment PET scans were recommended for the staging of all patients at all institutions throughout the study period; however, we could not verify the receipt of pretreatment PET scan because all institutions function as quaternary referral centers and occasionally had incomplete records regarding pretreatment staging and chemotherapeutic regimens. We initially included 94 consecutive patients treated between July 2008 and December 2017 with radiotherapy as a portion of their initial treatment for cHL. We excluded 3 patients who were lost to follow-up within 3 months of treatment completion. Risk stratification was determined based on the German Hodgkin Study Group criteria, with *unfavorable patients* defined as having any of the following risk factors: bulky disease, extranodal disease, involvement of 3 or more nodal areas, or a documented elevated erythrocyte sedimentation rate at diagnosis. We coded in-field versus out-of-field recurrences at the discretion of the treating physician after review of relevant computed tomography (CT) and PET imaging. All statistics were performed on JMP software (SAS Institute, Cary, North Carolina). The Kaplan-Meier statistics were used to provide estimates of overall survival and progression-free survival (PFS). The log-rank test statistic was used to assess any significant effect on those outcomes resulting from stratification by selected prognostic factors.

## Results

A total of 91 patients were included in the analysis. **Table 1** describes the patient- and treatment-related characteristics of our cohort. Most patients were female, had stage II disease, and did not present with B symptoms. The most common sites of disease were the cervical/supraclavicular areas and the mediastinal/hilar areas. Most patients received RT doses of ≥ 30 Gy (59%) and a slight majority received proton therapy (53%). The presence of unfavorable disease did not significantly affect the use of proton versus photon therapy (*P* = .13) or the selection of a dose > 30 Gy or < 30 Gy (*P* = .19).

**Table 1.** Patient, tumor, and treatment characteristics of adolescents and young adults treated for stage I/II classical Hodgkin lymphoma (N = 91).

**Variable**	**Patients, No. (%)**
Gender	
Female	58 (63.7)
Male	33 (36.3)
Age at diagnosis, y	
< 18	24 (26.4)
18 to < 30	34 (37.4)
30+	33 (36.3)
Stage	
I	12 (13.2)
II	79 (86.8)
B symptoms	
Yes	31 (34.1)
No	60 (65.9)
Location of disease at diagnosis	
Cervical/supraclavicular	78 (85.7)
Axillary	23 (25.3)
Mediastinal/hilar	74 (81.3)
Abdominal	5 (5.5)
Pelvic	2 (2.2)
Extranodal site	12 (13.2)
Radiation modality	
Photon	43 (47.3)
Proton	48 (52.7)
Radiation dose, Gy	
< 30	37 (40.1)
≥ 30	54 (59.3)

**Figure 1.**
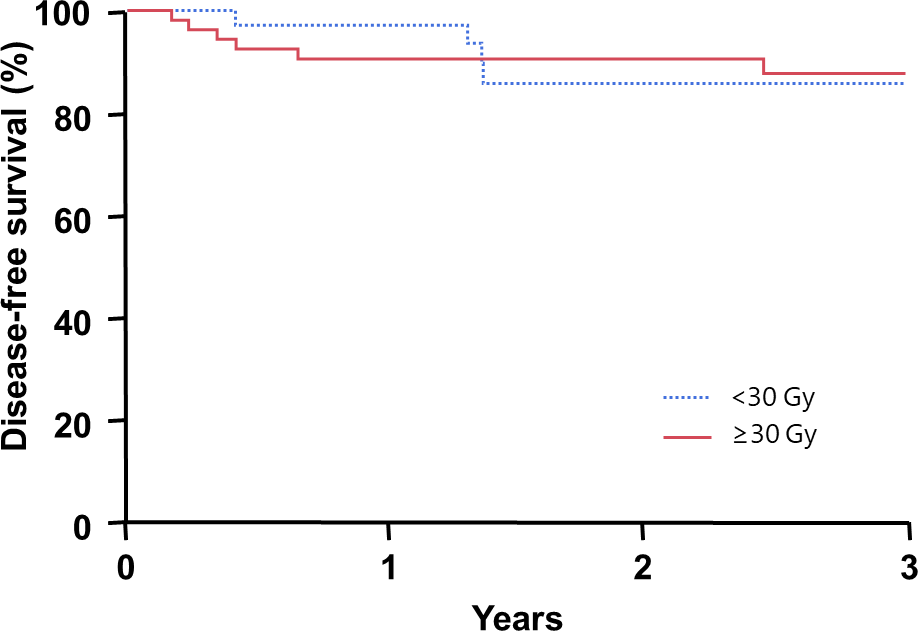
Kaplan-Meier plot of progression-free survival among adolescent and young adult patients distributed by total radiotherapy dose received.

**Figure 2.**
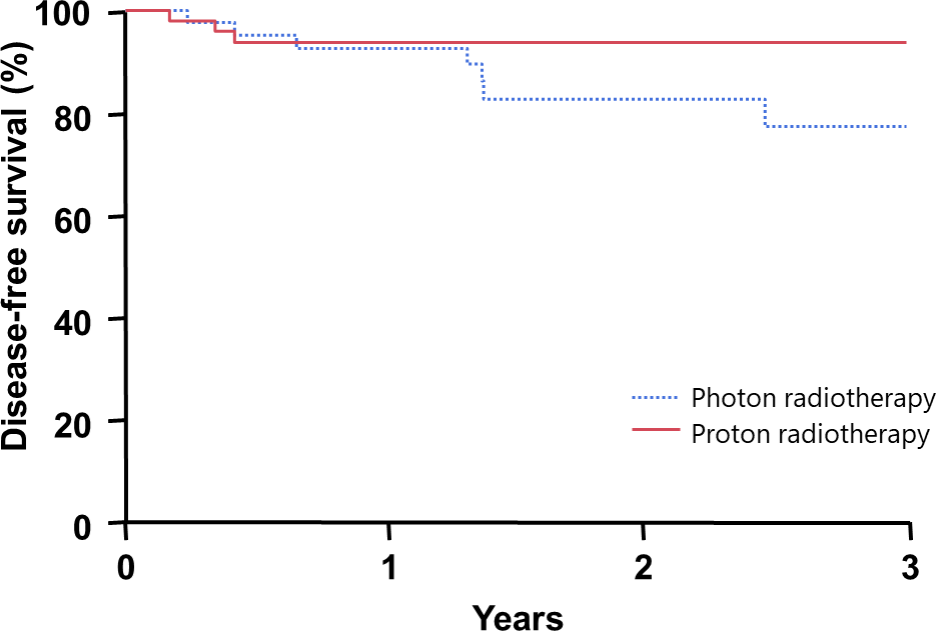
Kaplan-Meier plot of survival among adolescent and young adult patients by radiotherapy modality.

For the entire cohort, the 2-year overall survival rate was 98%, and the 2-year PFS rate was 89%. **Table 2** describes the patterns of failure in the 12 patients who experienced progression after RT by radiation dose and modality: 4 occurred out-of-field only, 2 occurred in-field only, and 6 experienced both in- and out-of-field progression. There was no significant difference in 2-year PFS among AYA patients by RT dose received (≥ 30 Gy, 91%; < 30 Gy, 86%; *P* = .82; **Figure 1**). Likewise, there was no difference in 2-year PFS among patients who received either proton or photon radiotherapy (proton, 94%; photon, 83%; *P* = .07; **Figure 2**). Patients receiving proton therapy were more likely to be female (75% versus 51%, *P* = .03), and to receive doses of RT of ≥ 30 Gy (81% versus 65%, *P* < .001). There were no significant differences by age, stage, or presence of B symptoms between patients treated with photon or proton RT. Of note, median follow-up was longer for patients who had proton (3.7 years) than photon (1.9 years) therapy. Patients with favorable, as compared to unfavorable disease, experienced similar 2-year PFS (94% versus 86%; *P* = .27).

**Table 2.** Patterns of failure by (a) radiation dose and (b) treatment modality in adolescents and young adults with stage I/II classical Hodgkin lymphoma (N = 91).

**Type of relapse**	**Patients, No. (%)**	**Patients, No. (%)**
Radiation dose	< 30 Gy (n = 37)	≥ 30 Gy (n = 54)
No relapse	33 (89.2)	46 (85.2)
In-field only	1 (2.7)	1 (1.9)
Out-of-field only	0 (0.0)	4 (7.4)
In- and out-of-field	3 (8.1)	3 (5.6)
Treatment modality	Proton therapy (n = 48)	Photon therapy (n = 43)
No relapse	44 (91.7)	35 (81.4)
In-field only	0 (0)	2 (4.7)
Out-of-field only	3 (6.2)	1 (2.3)
In- and out-of-field	1 (2.1)	5 (11.6)

## Discussion

These data demonstrate that AYAs with early stage cHL treated with RT have exceptional outcomes, consistent with prior studies and that those outcomes are excellent regardless of treatment protocol or RT dose used. We also show that outcomes are similar among patients treated with photon therapy and proton therapy. These findings can help inform future treatment discussions aimed at reducing the risk of radiation-induced late effects in this cohort of patients who have a very high chance of long-term survival at diagnosis.

The AHOD0031 trial [15] randomized pediatric patients with cHL with both a rapid early response to 2 cycles of ABVE-PC and a complete response following 4 cycles of ABVE-PC to either observation or 21 Gy of involved-field RT. Although no significant differences were seen in event-free survival with or without RT in the cohort, 88% of children who received RT and relapsed did so in an initially involved site of disease [15]. Among those receiving RT, AYA patients (those 15 years or older) were significantly more likely to experience relapse than children younger than 15 years at the time of diagnosis [16]. Those findings raise the concern that lower doses, such as 21 Gy, may be insufficient for adolescent patients. Those enrolled on AHOD0031 [15] would generally be considered unfavorable early stage cHL adult patients, with most modern protocols recommending 30 Gy of involved-site RT [5–7]. In a similar setting of exclusively adult patients with unfavorable early stage cHL enrolled in EORTC H10 [5] who were PET-negative after 2 cycles of ABVD (adriamycin, bleomycin, vinblastine, and dacarbazine) and subsequently received 2 further cycles of ABVD and 30 Gy of involved-node RT, only 69% of patients relapsed in an initially involved site of disease. In our analysis of solely AYA patients, however, there was no difference in PFS, and isolated in-field failures were rare, regardless of dose delivered. This suggests that lower doses are reasonable for adolescent patients being treated with pediatric systemic regimens and may reduce the late-effect burden in these patients.

Concerns over the late effects of RT in survivors of cHL have resulted in a decades-long effort to reduce the RT dose to healthy tissues at risk. Survivors of HL are at an increased risk of various late effects, among the most notable of which are cardiac disease, lung damage, and second malignancies [9–11]. All of these late effects are related to RT exposure in a dose-dependent manner [17–19]. Proton therapy can reduce the doses to organs at risk, especially in patients with mediastinal lymphomas [20, 21], thereby potentially reducing the life years lost to the late effects of RT [22]. The benefits of proton therapy are most pronounced in patients with lower mediastinal disease and female patients with axillary involvement [23]. Early reports have shown that proton therapy is associated with similar outcomes when compared with photon therapy for both adults and children with Hodgkin lymphoma [14, 24]. However, data comparing contemporaneous cohorts of patients treated with photon and proton therapy are sparse. We show that outcomes are similar between patients treated with proton therapy and photon RT, further supporting that, in patients for whom proton therapy results in significant dosimetric benefits to organs at risk, its use should be strongly considered.

There are limitations to our analysis that are inherent to any retrospective review. We cannot assess why patients were treated with the RT paradigm that was elected. Likewise, given trends suggesting a decreasing use of RT for early stage cHL, it is possible that patients treated more recently had worse baseline disease characteristics [25]. Further, given that we included patients from multiple institutions in this retrospective review, it is possible that there are inherent differences in the practice patterns and referral base demographics that could influence our results.

We present an analysis of AYA patients treated with radiotherapy for early stage cHL. We show that PFS is similar, regardless of total RT dose delivered or treatment modality used. We recommend the use of proton therapy in AYA patients for whom it provides a clear dosimetric benefit and is feasible to access.
